# Are all wines made from various grape varieties beneficial in the prevention of myocardial infarction and stroke?

**DOI:** 10.2144/fsoa-2020-0098

**Published:** 2020-11-09

**Authors:** Masahiro Iwasaki, Masahiro Murakami, Yoshinobu Ijiri, Muneshige Shimizu, Junichiro Yamamoto

**Affiliations:** 1 Division of Nutrition & Metabolism, Original Nutrition Co., Ltd, Osaka 532-0002, Japan; 2 Faculty of Pharmacy, Osaka Ohtani University, Osaka 584-8540, Japan; 3 Department of Health and Nutrition, Osaka Shoin Women's University, Osaka 577-8550, Japan; 4 Department of Fisheries, School of Marine Science and Technology, Tokai University, Shizuoka 424-8610, Japan; 5 Kobe Gakuin University (Professor Emeritus), Kobe 651-2180, Japan

**Keywords:** cardiovascular disease, fibrinolysis, grape, platelet aggregation, red wine, shear stress, stroke, thrombosis

## Abstract

**Aim:**

Epidemiologic studies support the assumption (French paradox hypothesis) that drinking red wine is beneficial in the prevention of cardiovascular diseases. Our recent works however cast doubt on such claim. Earlier we have shown that the antithrombotic activity of various fruits and vegetables mainly depends on their varieties. For this reason, several varieties of red and white grapes were tested for antithrombotic effect in animal experiments.

**Results:**

Antithrombotic effect of 45 red and white grape varieties were assessed in the present study. Out of the 45, one red grape variety showed antithrombotic effect, while the majority of red and white grape varieties enhanced thrombosis.

**Conclusion:**

Most red and white grape varieties enhanced thrombotic activity of blood.

## Lay abstract

Red wine is widely believed to prevent heart attack and stroke as claimed by a French Paradox hypothesis. Antithrombotic effect of 45 grape varieties was measured in the present study. Only very few red and white varieties inhibited the experimentally induced thrombosis while the majority of the tested varieties enhanced thrombotic activity of blood. Thus, our findings challenge the prevailing claims of the French Paradox.

Atherothrombosis plays important role in lifestyle-related myocardial infarction and stroke. Prevention of these diseases is an important social task in many countries. It has been shown that an appropriate diet may play an important role in the prevention of cardiovascular diseases. These studies have suggested that suitable intake of fruits and vegetable may prevent such diseases [1–5] and accordingly, many countries' health authorities have issued dietary guidelines [6–9]. Fruits and vegetables are classified into ‘healthy’ and ‘less healthy’ ones and they are recommended or advised against to their consumption accordingly [10].

Earlier we surveyed clinically useful blood tests for selecting fruits and vegetables for antithrombotic activity. Tests using anticoagulated blood have widely been used but they are not pathologically relevant and suitable for assessing thrombotic status of humans [11–13]. Blood tests using nonanticoagulated (native) blood and inducing thrombus formation solely by shear forces were developed in 1970 [14–21]. Recently, a commercially available point-of-care thrombosis test was shown to have pathological relevance to human arterial thrombosis [22–40].

Death from cardiovascular diseases in French people, who are taking thrombogenic high fat diet is surprisingly lower than in other European countries, although the latter take high-fat diet. Renaud and colleagues proposed that this may be due to the popular wine consumption of the French people (French paradox) [41]. Folts and colleagues demonstrated that red wine prepared from a special grape variety and the Welch's natural purple grape juice inhibited thrombus formation in animals (*in vivo*) and suggested that this may be due to some molecules such as polyphenols [42–45].

The aim of the present study was to test large number of grape varieties by a shear-induced thrombosis/thrombolysis test (Global Thrombosis Test) in order to assess the dependence of antithrombotic effect on the tested grape-varieties.

## Materials & methods

### Animals

13-week-old male Wistar ST rats (Japan SLC Co. Ltd, Hamamatsu, Japan) and 10-week-old male C57BL/6 mice (Japan SLC Co. Ltd, Hamamatsu, Japan) were purchased 1 week before the scheduled experiments. Animals were maintained in compliance with the ‘Guiding Principles for the Care and Use of Animals in the field of Physiological Sciences,’ published by Physiological Society of Japan. The protocol was approved by the Animal Experiment Committee of Kobe Gakuin University.

### Grapes

Eighteen grape varieties, (red) Asama merlot, Gamay noir, Cabernet sauvignon (Man), Cabernet franc, Syrah, Zenkoji, Pinot noir, Muscat bailey A (Man), Merlot, (white) Kerner, Shinano riesling, Chardonnay (Man), Sylvaner, Semillon, Sauvignon blanc, Pinot blanc, Muller thurgau, Riesling were donated from Manns wine Co., Ltd Japan; four varieties, (red) Cabernet sauvignon (Mer), Koshu, Muscat bailey A (Mer) and (white) Chardonnay (Mer) from Mercian Co. Ltd Yamanasi, Japan and twenty three varieties, (red) Aki queen, Oriental star, Kaiji, Charbono, Campbell early, Kyoho, Concord, Sunny rouge, Delaware, North red, Honey seedless, Honey black, Pione, Muscat bailey A (Nat), (white) Italia, Onsolo, Shine muscat, Dearing, Neo muscat, Honey venus, Pannonia kincse, Muscat of Alexandria, Megumi from National agriculture and food research organization.

## Preparation of grape juice filtrate

Several clusters of grapes were collected from various portions of one tree and from several trees to avoid deviation. Grapes with skin were washed crushed in a mortar at room temperature. The juice was centrifuged at 3000 rpm for 15 min at 4°C. The supernatant was filtered (pore size 5 μm, FP 30/5.0 CN-S, BM Instrument Co. Ltd, Japan) and the clear filtrates were stored at -80°C until use.

## *In vitro* assessment of platelet rich thrombotic & endogenous thrombolytic activities by the Global Thrombosis Test

The Global Thrombosis Test (GTT) (Thromboquest Ltd, London, UK) as described in detail earlier [46–49] was used to assess the antithrombotic effect of various grapes. The principle of this test had been described. Briefly, when nonanticoagulated blood flows through narrow channels, platelets are activated by high shear forces. Downstream from this point, the slow rate and turbulent flow favors the shear-activated platelets to aggregate and stabilized by the formed fibrin. Subsequently, the fibrin-stabilized platelet aggregates (thrombi) occlude the exit channels, causing the blood flow to be arrested. The instrument detects the time interval (d, s) between consecutive blood drops. At the start, blood flow is rapid and hence (d) is small. Subsequently, the flow rate decreases and hence (d) increases. When the actual (d) exceeds the default 15 s, the instrument displays ‘Occlusion Time (OT, s).’ Subsequently, the flow is restored due to thrombolysis, and is indicated by the detection of the first blood drop as ‘Lysis Time (LT, s).’ In the present measurements, blood was withdrawn from the abdominal aorta of rats 30 min after Nembutal anesthesia (pentobarbital sodium, 60 mg/kg, i.m.) and diluted with saline in 1:1 ratio. 0.4 ml grape filtrate or 0.4 ml saline (control) were added to 3.6 ml of the two-times saline-diluted blood, mixed and transferred to the GTT test tube within 15 s following the withdrawal of blood. Four tubes containing various concentrations of grape filtrates and one control were assessed simultaneously in the four-channel instrument and the tests were repeated six times for each sample (n = 6). Prolonged OT suggests inhibition while shortened OT indicates enhanced thrombus formation, respectively. Prolonged LT suggests inhibited while shortened LT indicates enhanced thrombolysis, respectively. In case that the filtrate added to blood prolongs OT and shortens LT compared with saline, the result was thought to predict that filtrate has antithrombotic activity. In case that the filtrate prolongs OT but gives no effect on LT, the result was thought to predict that filtrate has antithrombotic activity. In case that the filtrate prolongs OT and prolongs LT, the result was thought not to predict the activity. This has to be determined by the *in vivo* test. In case that the filtrate shortens OT and prolongs LT, the result was thought to predict that the filtrate has prothrombotic activity. In case that the filtrate shortens OT but gives no effect on LT, the result was thought to predict that the filtrate has prothrombotic activity. In case that the filtrate shortens OT and shortens LT, the result was thought not to predict the activity. This has to be determined by the *in vivo* test. Thus, overall antithrombotic/prothrombotic activities are determined by the balance between the effects on thrombus formation and lysis. Finally, this is confirmed by the employed *in vivo* test, as described below.

## *In vivo* assessment of arterial thrombotic activity & endogenous thrombolytic activity by Helium-Neon laser-induced thrombosis in mice

The Helium-Neon laser-induced thrombosis method has been described in detail [46–50]. In brief, the left femoral artery of an anaesthetized mouse was exposed, and Evans blue dye was injected intravenously. The center of the exposed carotid artery was irradiated with the laser beam, and formation of thrombus at the site of irradiation was monitored and recorded on videotape. Images of the outlined thrombus were computer-analyzed at intervals of 10 s. The area of thrombus was delineated, and the thrombus mass was calculated by multiplying gray scale and area using Image J software (Image Processing and Analysis Java version 1.30, National Institutes of Health, Maryland, USA). Thrombotic status was expressed as the total sum of thrombus mass after the first 10 min of irradiation.

## *In vivo* assessment of antithrombotic activity of grape filtrates after oral administration

Procedure has been described earlier in detail. In brief, filtrates and controls (distilled water) (3.85 ml/kg body weight) were orally administered to animals through a gastric tube. A second dose using the same volume of filtrate or water (3.85 ml/kg body weight) was given 30 min later. Mice were anaesthetized and the laser-induced thrombosis experiments commenced 90 min after the second oral treatment. The antithrombotic or prothrombotic effects were assessed by measuring the volume of the thrombus mass. Compared with controls, reduced volume of thrombus mass indicated antithrombotic while increased volume suggested prothrombotic effect.

## Measurement of antioxidant activity

Antioxidant activity was measured by chemiluminometry (Luminometer AB-2200; ATTO Co. Ltd, Tokyo, Japan). The reaction mixtures were (A) positive control, (B) negative control and (C) sample mixture. For (A), 150 μl 100 mmol/l phosphate buffer and 60 μl xanthine oxidase solution (XOD; 0.1 m/ml; Sigma-Aldrich Co, Ltd, MO, USA) were kept at 37°C for 1 min. Ten microliters of 2-methyl-6-p-methoxyphenylethynylimidazopyrazione (MPEC) (ATTO Co, Ltd), 30 μl saline and 50 μl 3.6 mmol/l hypoxanthine were added and mixed for 1 min. Measurements were performed over 45 s at 37°C. For (B), XOD and saline were replaced with phosphate buffer and the test sample, respectively. For (C), 10 μl saline in (A) was replaced with the test sample. Antioxidant activity was expressed as units of superoxide dismutase (SOD, units/ml filtrate) using a standard curve. Five parallel measurements were obtained from each sample.

## Measurement of polyphenol content in grape varieties

Control and test sample mixtures were prepared in a similar manner to those described for the measurement of antioxidant activity. Chlorogenic acid (Sigma-Aldrich Co, Ltd) was used as the standard polyphenolic acid. A mixture consisting of 3.2 ml distilled water, 20 μl filtrate sample or the standard polyphenolic solutions, 200 μl Folin–Dennis reagent and 400 μl saturated Na_2_CO_3_ solution was kept at room temperature for 30 min and changes in absorbance at 760 nm were read using a spectrophotometer (Ubest-35; Jasco Co., Tokyo, Japan). The polyphenolic content was expressed as chlorogenic acid concentration. Triplicate measurements were obtained from each test sample.

## Statistical analysis

Analysis was performed by a statistical package software (Unistat Light 5.6, Unistat Ltd, London, UK). Occlusion time (OT: raw data) and lysis time (LT; logarithmic data) were analyzed by repeated ANOVA, followed by *post hoc*, Dunnett. Thrombus mass in the laser-induced experiments was analyzed by ANOVA, followed by post hoc, Dunnett. Correlations of polyphenol contents/antioxidant activity and OT/LT were analyzed by Pearson Correlation Test. Results were expressed as means ± SEM. p < 0.05 was considered as statistically significant.

## Results

### Thrombotic & thrombolytic activities of red & white grape varieties assessed by the GTT

**Figure 1.**
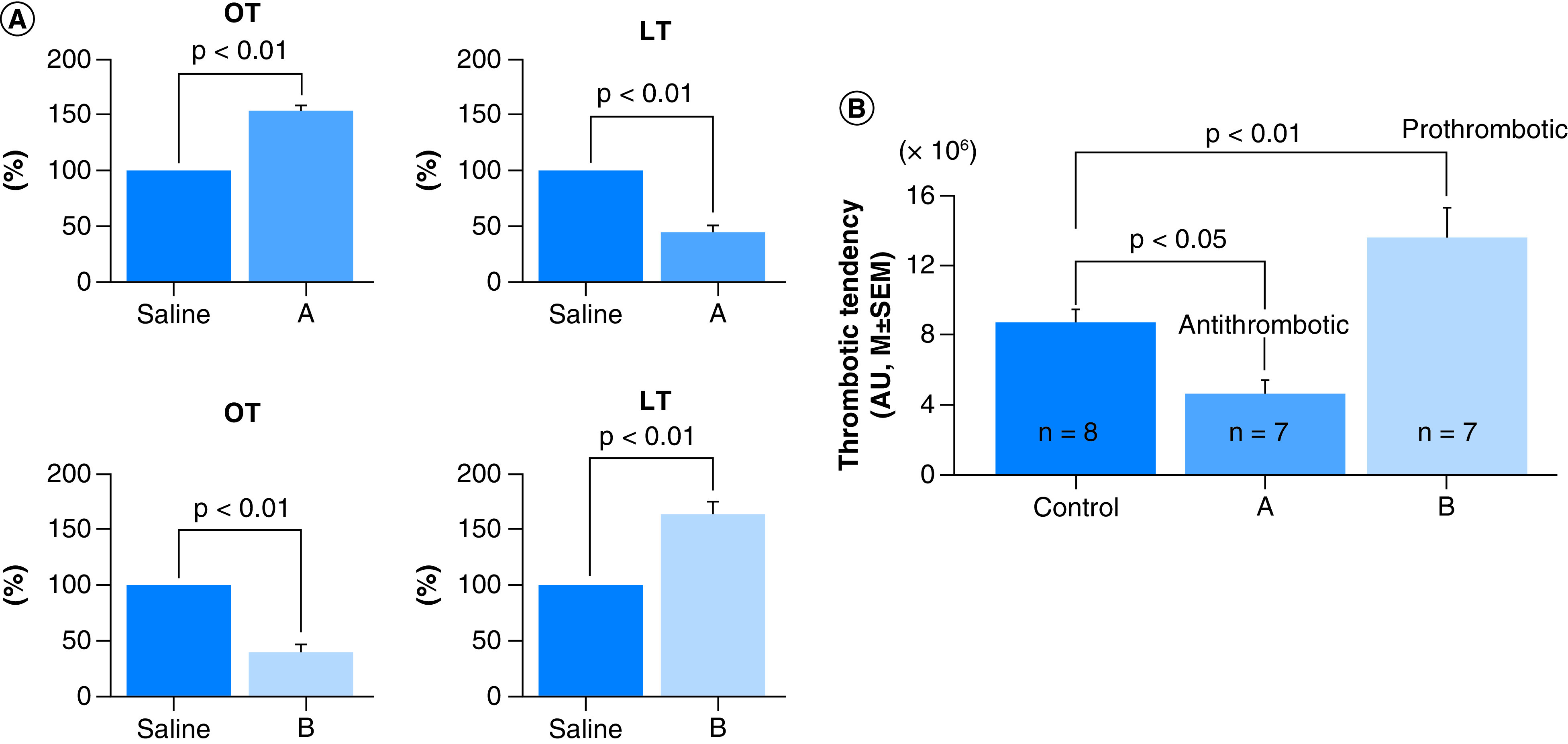
Effect of two grape varieties on thrombosis. **(A)** Comparison between antithrombotic activity of Cabernet sauvignon (Man) (A) and Neo muscat (B), assessed by the *in vitro* test (GTT). Mean values with standard error of the mean (SEM) are displayed. P-values are shown relative to saline (control). **(B)** Comparison of antithrombotic activity of Cabernet sauvignon (Man) (A) and Neo muscat (B), assessed by the *in vivo* test (laser-induced thrombosis test). Mean values with SEM are displayed. P-values are shown relative to saline (control). SEM: Standard error of the mean.

These activities and the predicted overall activities are shown in Table 1. OT was 304.5 ± 59.3 sec (Mean ± SD) and LT 615.7 ± 84.1 sec (Mean ± SD). As overall thrombotic status is determined by the balance between thrombotic (OT) and thrombolytic (LT) activities, predicted overall activities are shown in Table 1. Correlations between *in vitro* (GTT) and *in vivo* (laser-induced test) are shown in Figure 1. All varieties were classified in Table 3 according to the set criteria (Table 2). Measured with the GTT test, Cabernet sauvignon (Man) significantly inhibited thrombotic activity of blood (OT) and enhanced thrombolysis (LT), which suggest that this variety has significant antithrombotic effect. Neo muscat enhanced thrombotic activity of blood (OT) but inhibited thrombolysis (LT) suggesting an overall prothrombotic effect. These effects were confirmed by *in vivo* thrombosis test (Figure 1).

**Table 1.** Effect of grape varieties on thrombosis.

(A) Effect of red grape varieties on thrombotic and thrombolytic activities				
Grape variety	Dilution	Occlusion time (OT)	Lysis time (LT)	Predicted overall activity
Asama Merlot	x1	12 ± 6.9^‡^	105 ± 11.0	Prothrombotic
	x3	ND	ND	
	x10	91 ± 2.4	83 ± 4.0^‡^	
Aki queen	x1	22 ± 8.3^‡^	124 ± 24.5	Prothrombotic
	x3	ND	ND	
	x10	90 ± 2.6	86 ± 13.8	
Oriental star	x1	29 ± 5.7^‡^	391 ± 144.9	Prothrombotic
	x3	52 ± 9.1^‡^	147 ± 31.8	
	x10	88 ± 0.7	84 ± 7.4	
Kaiji	x1	81 ± 14.6	538 ± 163.9^‡^	Prothrombotic
	x3	75 ± 7.3	139 ± 33.7	
	x10	88 ± 1.4	105 ± 25.4	
Gamay noir	x1	58 ± 18.7^†^	110 ± 22.5	Prothrombotic
	x3	35 ± 17.9^†^	88 ± 15.8	
	x10	98 ± 3.0	77 ± 3.6	
Cabernet sauvignon (Man)	x1	151 ± 15.2^‡^	40 ± 6.0^‡^	Antithrombotic
	x3	ND	ND	
	x10	97 ± 5.2	81 ± 14.3	
Cabernet sauvignon (Mer)	x1	62 ± 9.8^†^	358 ± 112.6^†^	Prothrombotic
	x3	57 ± 17.0^†^	118 ± 16.9	
	x10	91 ± 6.3	81 ± 9.6	
Cabernet franc	x1	35 ± 8.3^‡^	60 ± 12.0	Prothrombotic
	x3	84 ± 0.6	65 ± 14.3	
	x10	118 ± 27.8	73 ± 9.1	
Campbell early	x1	26 ± 3.6^‡^	77 ± 6.6	Prothrombotic
	x3	39 ± 206^‡^	65 ± 608	
	x10	68 ± 17.0	76 ± 12.2	
Kyoho	x1	27 ± 7.9^‡^	119 ± 35.4	Prothrombotic
	x3	ND	ND	
	x10	86 ± 2.4	78 ± 18.5	
Koshu	x1	69 ± 6.5^‡^	418 ± 79.8^‡^	Prothrombotic
	x3	90 ± 3.2	222 ± 77.6	
	x10	95 ± 2.5	83 ± 7.9	
Concord	x1	83 ± 6.4^‡^	45 ± 10.3^‡^	not determined
	x3	95 ± 3.6	59 ± 3.6^†^	
	x10	90 ± 2.7	84 ± 10.0	
Sunny rouge	x1	48 ± 13.5^‡^	94 ± 17.7	Prothrombotic
	x3	ND	ND	
	x10	83 ± 1.0	105 ± 12.3	
Syrah	x1	40 ± 6.3^‡^	114 ± 23.8	Prothrombotic
	x3	15 ± 2.4^‡^	86 ± 6.7	
	x10	95 ± 3.2	151 ± 16.1	
Zenkoji	x1	55 ± 11.0^‡^	109 ± 18.2	Prothrombotic
	x3	ND	ND	
	x10	91 ± 3.5	90 ± 5.0	
Charbono	x1	100 ± 6.8	650 ± 130.4^‡^	Prothrombotic
	x3	89 ± 4.7	139 ± 7.0	
	x10	86 ± 1.7	118 ± 6.6	
Delaware	x1	ND	ND	Prothrombotic
	x3	ND	649 ± 47.7^‡^	
	x10	39 ± 1.6	76 ± 26.0	
North red	x1	17 ± 2.1^‡^	94 ± 29.6	Prothrombotic
	x3	14 ± 2.1^‡^	105 ± 7.6	
	x10	73 ± 14.6	90 ± 12.9	
Honey seedless	x1	ND	ND	Prothrombotic
	x3	ND	ND	
	x10	21 ± 5.8^‡^	117 ± 14.4	
Honey black	x1	27 ± 6.2^‡^	137 ± 9.1^†^	Prothrombotic
	x3	ND	ND	
	x10	88 ± 3.4	97 ± 4.0	
Pione	x1	23 ± 5.2^‡^	192 ± 47.1^‡^	Prothrombotic
	x3	ND	ND	
	x10	87 ± 2.9	83 ± 9.0	
Pinot noir	x1	23 ± 1.2^‡^	127 ± 12.8	Prothrombotic
	x3	23 ± 0.7^‡^	92 ± 11.7	
	x10	82 ± 1.3^†^	83 ± 7.9	
Muscat bailey A (Man)	x1	24 ± 9.3^‡^	61 ± 6.8^‡^	Not determined
	x3	ND	ND	
	x10	96 ± 6.3	91 ± 7.6	
Muscat bailey A (Mer)	x1	20 ± 4.2^‡^	171 ± 38.5^†^	Prothrombotic
	x3	35 ± 7.9^‡^	105 ± 12.4	
	x10	88 ± 1.2	107 ± 12.8	
Muscat bailey A (Nat)	x1	49 ± 13.4^‡^	255 ± 81.5	Prothrombotic
	x3	ND	ND	
	x10	83 ± 11.2^‡^	96 ± 6.5	
Merlot	x1	29 ± 5.8^‡^	87 ± 13.6	Prothrombotic
	x3	88 ± 20.4	98 ± 13.3	
	x10	101 ± 12.9	109 ± 17.0	
**(B) Effect of white grape varieties on thrombotic and thrombolytic activities**				
**Grape variety**	**Dilution**	**OT**	**LT**	**Predicted overall activity**
Italia	x1	52 ± 12.9^†^	255 ± 81.5^‡^	Prothrombotic
	x3	72 ± 2.7	128 ± 8.6	
	x10	89 ± 1.5	96 ± 6.5	
Onsolo	x1	118 ± 13.8	140 ± 47.7	No effect
	x3	84 ± 1.2	169 ± 575.2	
	x10	97 ± 6.9	273 ± 100.3	
Kerner	x1	21 ± 3.7^‡^	84 ± 17.4	Prothrombotic
	x3	37 ± 15.5^‡^	112 ± 12.6	
	x10	94 ± 2.4	101 ± 12.5	
Shinano riesling	x1	37 ± 8.7^‡^	77 ± 7.3	Prothrombotic
	x3	89 ± 1.1	86 ± 18.1	
	x10	96 ± 1.5	116 ± 38.1	
Shine muscat	x1	15 ± 2.1^‡^	128 ± 31.7	Prothrombotic
	x3	21 ± 0.8^‡^	119 ± 12.0	
	x10	75 ± 3.3	122 ± 25.9	
Chardonnay (Man)	x1	38 ± 5.1^‡^	162 ± 13.6^‡^	Prothrombotic
	x3	ND	ND	
	x10	79 ± 4.8	111 ± 9.7	
Chardonnay (Mer)	x1	33 ± 6.4^‡^	441 ± 176.5^†^	Prothrombotic
	x3	14 ± 2.8^‡^	158 ± 11.8	
	x10	87 ± 2.4	145 ± 70.9	
Sylvaner	x1	34 ± 6.9^‡^	132 ± 23.4	Prothrombotic
	x3	44 ± 17.0^‡^	102 ± 9.4	
	x10	98 ± 4.1	94 ± 14.0	
Sémillon	x1	36 ± 8.6^‡^	123 ± 21.6	Prothrombotic
	x3	ND	ND	
	x10	92 ± 4.7	88 ± 14.7	
Sauvignon blanc	x1	20 ± 0.2^‡^	183 ± 25.5	Prothrombotic
	x3	ND	ND	
	x10	8 ± 3.2^‡^	100 ± 18.3	
Dearing	x1	21 ± 3.5^‡^	493 ± 173.0	Prothrombotic
	x3	35 ± 9.7^‡^	609 ± 125.6^‡^	
	x10	103 ± 4.0	169 ± 65.5	
Neo muscat	x1	57 ± 5.7^‡^	679 ± 59.2^‡^	Prothrombotic
	x3	53 ± 8.8^‡^	144 ± 13.8^†^	
	x10	86 ± 3.3	124 ± 6.8	
Honey venus	x1	23 ± 4.5^‡^	77 ± 11.8^†^	Not determined
	x3	ND	ND	
	x10	85 ± 2.6	79 ± 4.6	
Pannonia kinsce	x1	28 ± 7.1^‡^	150 ± 27.2^†^	Prothrombotic
	x3	50 ± 13.4^‡^	113 ± 20.8	
	x10	89 ± 3.9	90 ± 5.2	
Pinot blanc	x1	48 ± 5.7^‡^	68 ± 16.5	Prothrombotic
	x3	ND	ND	
	x10	77 ± 6.8^‡^	96 ± 7.6	
Muscat of alexandria	x1	21 ± 3.5^‡^	417 ± 102.7^‡^	Prothrombotic
	x3	12 ± 1.0^‡^	143 ± 9.0	
	x10	87 ± 2.9	94 ± 4.3	
Megumi	x1	28 ± 4.3^‡^	127 ± 19.0	Prothrombotic
	x3	ND	ND	
	x10	89 ± 4.1	98 ± 6.0	
Riesling	x1	62 ± 16.2^‡^	95 ± 19.5	Prothrombotic
	x3	88 ± 2.9	86 ± 17.6	
	x10	98 ± 2.4	104 ± 8.4	
Müller thurgau	x1	22 ± 5.5^‡^	91 ± 11.5	Prothrombotic
	x3	ND	ND	
	x10	75 ± 9.1	99 ± 9.9	

Mean values (±SEM) are expressed relative to saline (control).

† p < 0.05.

‡ p < 0.01.

ND: Not determined.

**Table 2.** Criteria of classifying grape varieties

(A) Criteria of classifying grape varieties based on thrombotic activity (OT)				
Dilution factor	Occlusion time (OT)	Index	Occlusion time (OT)	Index
x1	Not measured by blood running out	+1	Not measured by blood coagulation	-1
x1	Significant increase	+1	Significant decrease	-1
x3	Significant increase	+2	Significant decrease	-2
x10	Significant increase	+3	Significant decrease	-3
>x10	Significant increase	+4	Significant decrease	-4
**(B) Criteria of classifying grape varieties based on thrombolytic activity (LT)**				
**Dilution factor**	**Lysis time (LT)**	**Index**	**Lysis time (LT)**	**Index**
x1	Not measured by blood running out	+1	Not measured by blood coagulaton	-1
x1	Significant decrease	+1	Significant increase	-1
x3	Significant decrease	+2	Significant increase	-2
x10	Significant decrease	+3	Significant increase	-3
>x10	Significant decrease	+4	Significant increase	-4

Juice (x1) was diluted with saline, then mixed with diluted nonanticoagulated blood and measured.

**Table 3.** Antithrombotic activity ranking of grape varieties.

(A) Antithrombotic activity ranking of red grape varieties based on thrombotic activity (OT)	
Intensity	Varieties
+2	
+1	Cabernet sauvignon (Man)
0	Kaiji, Charbono
-1	Asama merlot, Cabernet franc, Zenkoji, Muscat bailey A (Man), Merlot, Aki queen, Kyoho, Concord, Sunny rouge, Delaware, Honey seedless, Honey black, Pione, Koshu
-2	Gamay noir, Syrah, Oriental star, Campbell early, North red, Cabernet sauvignon (Mer), Muscat bailey A (Mer)
-3	PiNot noir, Muscat bailey A (Nat)
ND	
**(B) Antithrombotic activity ranking of white grape varieties based on thrombotic activity (OT)**	
**Intensity**	**Varieties**
+1	
0	Onsolo
-1	Shinano riesling, Chardonnay (Man), Sémillon, Riesling, Müller thurgau, Italia, Honey venus, Pannonia kincse, Megumi
-2	Kerner, Sylvaner, Shine muscat, Dearing, Neo muscat, Muscat of Alexandria, Chardonnay (Mer)
-3	Pinot blanc, Sauvignon branc
-4	
**(C) Antithrombotic activity ranking of red grape varieties based on thrombolytic activity (LT)**	
**Intensity**	**Varieties**
+2	Concord
+1	Cabernet sauvignon (Man), Muscat bailey A (Man)
0	Gamay noir, Cabernet franc, Syrah, Zenkoji, Pinot noir, Merlot, Oriental star, Campbell early, Kyoho, Sunny rouge, North red, Aki queen
-1	Kaiji, Charbono, Delaware, Honey seedless, Honey black, Pione, Cabernet sauvignon (Mer), Koshu, Muscat bailey A (Mer)
-2	
ND	Asama merlot, Muscat bailey A (Nat)
**(D) Antithrombotic activity ranking of white grape varieties based on thrombolytic activity (LT)**	
**Intensity**	**Varieties**
+2	
+1	Honey venus
0	Kerner, Shinano riesling, Sylvaner, Sémillon, Sauvignon branc, Megumi, Pinot blanc, Müller thurgau, Riesling, Onsolo, Shine muscat
-1	Chardonnay (Man), Chardonnay (Mer), Italia, Pannonia kincse, Muscat of Alexandria
-2	Neo muscat
-3	
ND	Dearing

LT: Lysis time; OT: Occlusion time.

Cabernet sauvignon (Man) was classified as an antithrombotic grape. Concord, Muscat bailey A (Man) and Honey venus did not show such effect. Most of the red and white grape varieties were prothrombotic.

### Effect of heat treatment on thrombotic & thrombolytic activities

Heat stability of eight varieties was assessed after their treatment at 100°C for 10 min. The treatment changed prothrombotic activity to antithrombotic only in Cabernet franc but in other varieties heat treatment did not affect their thrombotic activities. Results are shown in Table 4.

**Table 4.** Effect of heating on thrombotic and thrombolytic activities.

Grape variety	Heat treatment	Occlusion time (OT)	Lysis time (LT)	Predicted overall activity
Cabernet sauvignon (Man)	Before	127 ± 14.3^†^	74 ± 15.7^†^	Antithrombotic
	After	88 ± 2.5	34 ± 4.7^‡^	Less antithrombotic
Onsolo	Before	121 ± 18.4	201 ± 89.0	No effect
	After	113 ± 9.4	386 ± 116.5	No effect
Cabernet franc	Before	44 ± 20.7^†^	55 ± 6.2	Prothrombotic
	After	125 ± 89.8	47 ± 13.2^†^	antithrombotic
Neo muscat	Before	55 ± 10.3^‡^	505 ± 56.3^‡^	Prothrombotic
	After	30 ± 5.3^‡^	370 ± 68.8^‡^	Prothrombotic
Pinot blanc	Before	30 ± 4.7^‡^	97 ± 11.1	Prothrombotic
	After	31 ± 5.1^‡^	80 ± 12.8	Prothrombotic
Koshu	Before	61 ± 6.1^†^	461 ± 104.5^‡^	Prothrombotic
	After	49 ± 4.1^†^	239 ± 113.9	Prothrombotic
Müller thurgau	Before	30 ± 9.7^‡^	91 ± 16.5	Prothrombotic
	After	54 ± 21.1^‡^	99 ± 17.4	Prothrombotic
Shine musca	Before	10 ± 1.9^‡^	57 ± 10.9	Prothrombotic
	After	10 ± 0.3^‡^	82 ± 26.8	Prothrombotic

Filtrates were heated for 10 min at 100°C and thrombotic and thrombolytic activities were measured by GTT. Values are expressed as relative ones to saline.

† p < 0.05.

‡ p < 0.01.

ND: Not determined.

### Polyphenol content in red & white grape varieties

**Figure 2.**
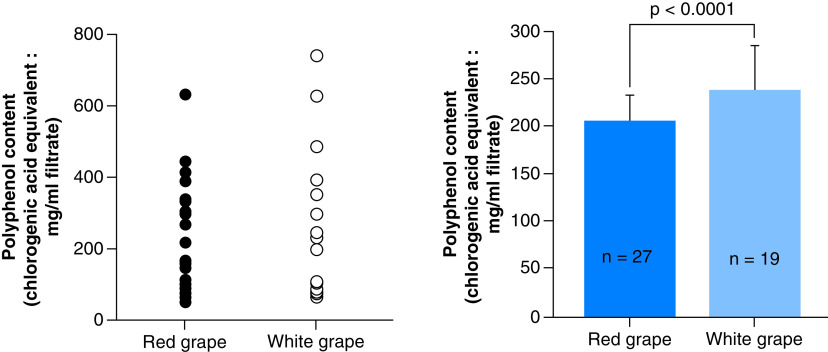
Polyphenol content in red and white grape varieties. Mean values with SEM (error bars) are displayed.

Results are shown in Figure 2. Polyphenol contents were very diverse both in red and white grape varieties and the content in red grape varieties was significantly lower than that in white grape varieties.

### Correlation between polyphenol content/antioxidant activities & thrombotic/thrombolytic activities

**Figure 3.**
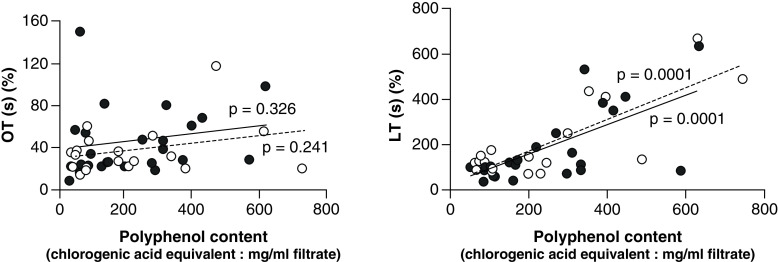
Correlation between polyphenol content and thrombotic (OT)/thrombolytic (LT) activities. P-values in OT in red and white varieties are 0.326 and 0.241, respectively; p-Values in LT in red and white varieties are 0.0001 and 0.0001, respectively. LT: Lysis time; OT: Occlusion time.

Polyphenol content of red and white grape varieties is shown in Figure 2. The contents were diverse in red and white varieties (Figure 2). The content in white grape varieties was significantly higher than that in red grape varieties. Correlation between polyphenol content and thrombotic/thrombolytic activities obtained by the *in vitro* test (GTT) was analyzed (Figure 3). No significant correlation was shown between OT and polyphenol content. In contrast, significant correlation was shown between LT and polyphenol content. This suggests that polyphenol enhances overall thrombotic activity both in red and white grape varieties. No significant correlation was found between antioxidant activity and thrombotic/thrombolytic activities.

## Discussion

Since the proposed French paradox hypothesis and findings of various epidemiologic studies, the prevailing idea was that consumption of any red wine is beneficial in the prevention of cardiovascular diseases. Experimental findings using animal models of thrombosis supported such claim [42–45]. These experiments showed that intravenously and orally administered red wine and purple grape juice prepared from some special grape variety inhibited thrombosis *in vivo*. Similar findings were reported when anticoagulated blood samples were tested *in vitro* by agonist-induced platelet aggregation tests.

Pathologically relevant point-of-care thrombosis tests were not commercially available for some time. Platelet function (thrombosis) tests commonly in use are testing anticoagulated blood. Flow and shear forces, main determinants of thrombus formation *in vivo* were not present in these tests. Currently much attention has been focused on a point-of-care shear-induced thrombosis/thrombolysis *in vitro* test (GTT), which is using for the first time nonanticoagulated blood.

A total of 26 red grape and 19 white grape varieties were tested for antithrombotic effect by the GTT test. This technique can measure not only thrombotic and thrombolytic/fibrinolytic activities but also predict the overall antithrombotic activity [46–48]. This was also confirmed in the present study (Figure 1). Tested by this technique, one red grape variety Cabernet sauvignon (Man) showed overall antithrombotic activity but the overall antithrombotic effect of two varieties, Concord and Muscat bailey A (Man), was not certain. The latter should be investigated in the future by *in vivo* tests. All other varieties had no inhibitory effect on thrombus formation, rather they were prothrombotic. In white grape varieties, Honey venus might be antithrombotic but this needs to be confirmed by using *in vivo* tests. Majority of tested white grape varieties were prothrombotic. All varieties were classified as shown in Table 3 according to the criteria shown in Table 2. The inconsistency between Cabernet sauvignon (Man) and Cabernet sauvignon (Mer) might derive from their suppliers or vine trees. This problem remains to be solved in the future.

Heat stability of the experimental antithrombotic effect are shown in Table 4. The effect of most varieties was heat stable but in Cabernet franc the thrombotic activity changed from prothrombotic to antithrombotic by heat treatment.

Folts and colleagues suggested that antithrombotic activity may be due to polyphenol and antioxidant content of wines [42–45]. For this reason, in the present study the relationship between GTT-measured thrombotic (OT)/thrombolytic activities (LT) and polyphenol content/antioxidant activity of grape varieties were also investigated. Polyphenol content had no significant relation to the effect on thrombus formation, but it had an inhibition on thrombolysis.

In this study we selected the chlorogenic acid as representative of polyphenolic acids on the grounds that quite a few published studies did the same. However, it should be considered that chlorogenic acid were not found in grapes but in coffee beans. Our findings, that chlorogenic acid contents significantly relate to the thrombolytic activity of grape extract may stimulate future investigation of using polyphenolic acid representative other the chlorogenic acid to clarify this issue.

The antioxidant activity of grapes had no significant relation to either thrombosis or thrombolysis.

In an earlier study of mulberry, it was found that antioxidants had inhibitory effect on thrombosis [46]. Polyphenols inhibited thrombosis in carrots [47]. Antioxidant activity and polyphenol content had no significant relation to thrombosis (OT) and thrombolysis (LT) in apple [48]. Lycopene had no significant relation to the antithrombotic activity [49]. These inconsistencies together with the present findings do not support the assumption that these components determine the antithrombotic activity of fruits and vegetables. This is however not inconsistent with findings that certain purified components had antithrombotic effects [51–54]. We have to pay attention to the finding that filtrates of different grape varieties are equivalent to the different wines. Simple phenols might be transformed into complex molecules during wine production and wine aging. Moreover, by filtration of grapes only compounds present in the pulp are extracted, but not polyphenols which are present in the skin and seeds, which can be extracted only by maceration during wine making. However, the assumption that all red wine is antithrombotic should be put to further investigation.

We have selected fruit and vegetable varieties with experimental antithrombotic effect [55–60] and demonstrated that oral intake of these varieties can inhibit thrombotic status in humans [61,62].

## Conclusion

The aim of the present study was to determine whether only red wines and not white wines have antithrombotic effect. In this study grape juices prepared from various red and white grape varieties were tested by pathologically relevant *in vitro* and *in vivo* thrombosis test. The study demonstrated that both red and white grape juices obtained from various varieties can have antithrombotic effect and such effect is dependent on the variety of tested grape. As such, the present findings challenge the French paradox hypothesis and put it into a new light.

## Future perspective

According to the ‘French paradox’ the popular consumption of red wines, antithrombotic effect is solely responsible for the low incidents of coronary heart disease and stroke in the French population. The present study confirms this assumption but found some white wines also having antithrombotic effect. Thus, wines from special varieties of grapes, together with intake of antithrombotic fruit and vegetable varieties may constitute an antithrombotic diet, effective in the prevention of myocardial infarction or stroke.

**Summary points**

- Antithrombotic activity of 26 red grape varieties and nineteen white grape varieties were measured by an *in vitro* test which use nonanticoagulated blood (Global Thrombosis Test), and also by an animal model of thrombosis.
- In general, the antithrombotic effect or the lack of it depended on the variety of the tested grapes.
- Both red and white wine from specific varieties may have antithrombotic effect, while the majority of wines do not have significant effect on thrombotic activity of blood.
- Our findings put the French paradox hypothesis into new light.
